# “I think I can”: achievement-oriented themes in storybooks from Indonesia, Japan, and the United States

**DOI:** 10.3389/fpsyg.2014.00167

**Published:** 2014-03-04

**Authors:** Maria Suprawati, Florencia K. Anggoro, Danuta Bukatko

**Affiliations:** ^1^Fakultas Psikologi, Universitas Sanata DharmaYogyakarta, Indonesia; ^2^Department of Psychology, College of the Holy CrossWorcester, MA, USA

**Keywords:** achievement, storybooks, preschoolers, culture, socialization, Japan, Indonesia

## Abstract

The focus of the present study is on the ways in which storybooks communicate cultural ideals about achievement orientation, and in particular, the role of effort, perseverance, and hard work in fostering successful outcomes. Sixty preschool children's books from Indonesia, Japan, and the United States (20 from each country) were examined for the presence of achievement-oriented themes. These countries were chosen due to previously documented cultural differences in models of learning and individualist/collectivist tendencies that could have some bearing on achievement outcomes. Texts were assessed for (1) the frequency with which “challenge events” appeared in the narratives, (2) whether these events derived from sources internal or external to the main character, and (3) whether solutions relied on the main character individually or included the assistance of others. Results show that Japanese storybooks contained significantly more challenge events than Indonesian storybooks. Compared with Japanese storybooks, American storybooks tended to include a greater proportion of challenges derived from internal qualities of the main character as opposed to external factors. Compared with American storybooks, Japanese storybooks contained a significantly greater proportion of challenges that were solved with individual efforts as opposed to efforts involving the assistance of others. Findings from this study contribute to our understanding of how storybook contexts can provide a rich source of information for young children learning about culturally valued qualities and behaviors related to achievement.

## Introduction

The values and beliefs children bring to the school experience have important consequences for their success. For example, students who have *perceived control*—that is, they believe that they can influence success or failure in school, tend to achieve higher grades, primarily through their greater engagement with classroom activities (Skinner et al., 1990). Similarly, research shows that *self-efficacy*, the individual's belief in her or his capabilities to attain specific goals, plays a role in academic achievement (Bandura, 1997; Zimmerman, 2000). Still another line of research shows that students who hold *incremental* (or malleable) *theories* of their intelligence choose more effort-based strategies in response to classroom failures and obtain higher grades than students who believe intelligence is fixed (Elliot et al., 1999; Blackwell et al., 2007). Children show different profiles in their approach to challenging tasks as early as kindergarten, with some displaying the belief that success comes from trying hard, while others exhibit feelings of lack of control (Ziegert et al., 2001). Thus, in examining the factors that are related to high vs. low achievement in school, it is important to consider how children form beliefs about themselves as learners and to identify the ways in which children's experiences convey information about what it takes to be successful even before they enter the formal educational system.

The focus of the present study is on the ways in which storybooks communicate cultural ideals about achievement orientation, and in particular, the role of effort, perseverance, and hard work in fostering successful outcomes. These ideals take on special significance in light of accumulating evidence that self-regulation, delay of gratification, and persistence are among the strongest predictors of academic success as children progress through the educational system (Duckworth and Seligman, 2005; Duckworth et al., 2007).

Storybooks targeted for preschoolers can be viewed as cultural tools that contain a wealth of information about social norms, values, and personal traits that are desirable within societal groups. As Lamoreaux and Morling (2012) maintain, there is value in examining cultural products since they likely reflect the “psychologies of members of a cultural group.” For example, storybooks for preschoolers have been identified as sources of information for children about the specific emotions that are valued in different cultures (Tsai et al., 2007), as well as the mental states that guide how children understand themselves and others (Dyer et al., 2000; Dyer-Seymour et al., 2004). In the case of understanding the development of achievement motivation and beliefs, it may be that the narratives children encounter in storybooks offer information about the ingredients for successful problem resolution, and especially the personal qualities and behaviors that are linked to achieving some goal or objective successfully. That is, storybooks may be important complements to the messages children receive from caregivers and other sources about behaviors and beliefs necessary for success.

In the present study, 60 preschool children's books from Indonesia, Japan, and the United States (20 from each country) were examined for the presence of achievement-oriented themes. These countries were chosen due to observed cultural differences in models of learning and individualist/collectivist tendencies that could have some bearing on achievement outcomes. Previous research has pointed to the tendency for Japanese students to attribute success to hard work and failure to lack of effort, in contrast to Anglo American children, who attribute success to a range of factors including luck and ability along with effort (Holloway, 1988). Furthermore, such cross-cultural differences in beliefs about effort are enacted in divergent patterns of responses to failure. Heine et al. (2001) reported that Japanese students who failed a problem-solving task responded by persisting even more, in contrast to North American students, who were less likely to persevere. It is possible that parental values and parenting practices have an influence on these patterns in children's achievement beliefs. As Holloway et al. (1986) have reported, Japanese mothers see failures in children's performance as primarily due to lack of effort, whereas American mothers view children's failures as due to a mix of low effort and low ability. However, as important as they are, parental “inputs” about qualities related to achievement are likely to be only one force shaping children's notions about culturally-valued qualities of the self. Our intent was to examine how variations in cultural products such as storybooks may also play a part in this process.

There is little research on achievement beliefs among students in Indonesia. Liem and Nie (2008) found that secondary students in Indonesia tended to hold more performance-oriented than mastery-oriented goals, being more attuned to achieving externally determined goals in a way that is socially approved than to intrinsic motivations to learn when compared to Chinese students. When we consider Dweck's (1999) research on achievement motivation, the implication is that for these students, there might be less value placed on the role of effort. The inclusion of storybooks from Indonesia offers an opportunity to see how a community that shares an orientation to collectivism with Japan, but which may differ in other aspects concerning notions of the self that may be shared with the United States, portrays messages about achievement to young children. Heine (2001) suggests that many aspects of the East Asian self (Japan), characterized by collectivism, interdependence, and a focus in interpersonal harmony, can be understood as arising from principles of Confucianism. Since this tradition emphasizes the importance of changing the self in order to accommodate to the demands of the social world, the prominence of effort beliefs in Japan seems like a natural consequence. Indonesia is typically considered a collectivist society (Hofstede, 1991). However, its religious tradition is considerably different from other East Asian countries. While Confucianism is recognized as one of Indonesia's six official religions, less than 0.2% of the population now self-reports as belonging to this group (Sensus Penduduk, 2010). The implication is that perhaps beliefs about effort might take a different form in this country.

The storybooks we selected were intended to represent widely read classic and contemporary works targeted for preschool children ages 3–4 in Indonesia, Japan, and the United States. In analyzing the content of these storybooks, we conceptualized achievement themes as being manifest by narratives in which the principal character encountered challenges or obstacles that had to be overcome. Texts were assessed for the frequency with which “challenge events” appeared in the narratives, the idea being that repeated attempts to solve a given problem or successive problems provided a message about the importance of effort, perseverance, and trying again without giving up. In an attempt to capture cultural variations concerning individual vs. collectivist themes, we also assessed whether these challenge events derived from sources internal or external to the main character, and whether solutions to challenge events relied on the individual actions or resources of the main character or whether the main character benefited from the assistance of others.

## Materials and methods

A sample of 60 narrative storybooks from the United States, Japan, and Indonesia (20 per country) targeted for 3- to 4-year-olds was included in the study (see Supplemental Material for a complete list). Books from the United States and Japan were chosen using guidelines from previous research by Dyer et al. (2000); Dyer-Seymour et al. (2004). These books were selected from a larger database of approximately 190 children's storybooks compiled by Japanese experts and over 350 books compiled by American experts. These books were frequently read to young children and deemed appropriate for 3- to 4-year-olds based on the researchers' consultations of guidebooks for Japanese and American parents (Dyer-Seymour et al., 2004). Given a lack of comparable guidelines in Indonesia, the Indonesian storybooks were selected through teacher recommendations from 7 preschools and kindergartens in three cities in Central Java: Yogyakarta, Muntilan, and Semarang. We then equated the reading level of these books by computing the word density (number of words per page) of each American book and choosing 20 Indonesian books with comparable density.

### Coding

All of the storybooks in the study sample were written in the original languages (English for books from the United States, Japanese for books from Japan, and Indonesian for books from Indonesia). In order to avoid confounds involving the coders' native language, their countries of origin, and personal values regarding effort and achievement, we decided to have the books from Japan and Indonesia translated into English and have a single, native English-speaking coder (who was blind to our hypotheses) complete the coding of all books. The translators were Japanese-English and Indonesian-English bilingual speakers who were also blind to the study hypotheses.

In each storybook, our analysis focused on the main character, which was determined by several criteria. First, the main character is usually stated in the title (e.g., “the Runaway Bunny”). Second, the main character is mentioned or described more than other characters in the book. Third, the main “character” could be considered to be more than one individual (e.g., a team or pair of individuals), but only when there is no more detailed description of one character over another, or no one individual is mentioned more than another. Finally, the main character could be an entire group of individuals, as long as these individuals are mentioned as a unit.

Once the main character was established, we identified challenge episodes. A *challenge episode* is where the main character encounters an obstacle or difficulty that could prevent him/her from achieving a goal. We then coded each challenge episode along several dimensions. First, we determined the *source* of the challenge: Internal or External. An Internal source is where the challenge comes from some quality or behavior of the main character him/herself. For example, *Betsy stopped singing because she got sick; Michael decided not to join his friends on the basketball court because he believes he is too small*. An External source is when the challenge comes from the environment, a situation, or other characters outside of the main character. For example, *Betsy stopped singing because her parents didn't allow her to sing anymore; Michael didn't play basketball because someone took his ball*. Second, we identified the *solution* for the challenge: Individual or Social. An Individual solution is when the main character overcomes the challenge by her/himself. A Social solution is when the main character receives help from another character to overcome the challenge. Finally, at the end of each book, we determined whether the main character achieves his/her goal (Success or Failure). If the main character is successful, we also identified who gets the benefit of the solution in the end: only the main character (Individual benefit), an(other) character(s) (Other benefit), or both (Shared benefit). To assess the reliability of this coding, another native English-speaking coder (also blind to our hypotheses) scored a randomly selected set of 10 American storybooks in the sample. The inter-rater reliability between these coders was 0.78.

## Results

Based on the coding scheme described above, we analyzed the data by comparing the three countries in terms of the number of challenge events, the sources/types of challenges and their solutions, and the nature of the overall outcome.

### Book lengths

To examine whether the lengths of the books were equivalent for the three countries in the sample, we counted the total number of sentences for each book from Indonesia, Japan, and the United States. A One-Way ANOVA on this measure showed a significant effect of country, *F*_(2, 59)_ = 7.324, *p* = 0.001. *Post-hoc* analyses indicated that Japanese and American storybooks did not differ significantly from each other in length (*M*_Japanese_ = 52.55, *SD* = 18.71; *M*_American_ = 56.2, *SD* = 28.65). However, Indonesian storybooks were significantly lower in length compared to storybooks from Japan and the United States (*M*_Indonesian_ = 31.05, *SD* = 18.53, *p* = 0.010, and *p* = 0.002, respectively).

### Number of challenge events

For each book, we tallied the number of challenge events present in the textual information in the narratives. Most of the books in our sample included at least one challenge event (85% of the Indonesian books, 90% of the Japanese books, and 80% of the American books). A One-Way ANOVA conducted on the total number of challenge events as a function of country yielded a significant effect of country, *F*_(2, 59)_ = 3.91, *p* = 0.026. *Post-hoc* analyses showed that Japanese books had significantly more challenge events than Indonesian books (*M*_Japanese_ = 4.75, *SD* = 2.49; *M*_Indonesian_ = 2.6, *SD* = 1.76, *p* = 0.035) and that American books had a greater number of challenge events than Indonesian books, although the difference was only marginally significant (*M*_American_ = 4.5, *SD* = 3.46, *p* = 0.07). There was no significant difference in the mean number of challenge events for Japanese and American books.

These findings, of course, need to be tempered by the fact that book lengths for the Indonesian sample were shorter compared to Japan and the United States. There is less opportunity for challenge events to appear if books have less content. However, if the Indonesian books can be considered to represent the typical storybook experience for preschool children in that country, these data suggest that Indonesian children receive less exposure to characters that repeatedly attempt to overcome obstacles than children in Japan and the United States.

### Types of challenges and solutions

In order to obtain a better understanding of the types of challenges encountered by characters in the storybooks, we tallied the proportion of challenge events that were coded as Internal to the main character in their origin for each book. A One-Way ANOVA on this measure indicated a marginally significant effect of country, *F*_(2, 59)_ = 2.52, *p* = 0.089. American books depicted more Internal challenge events (*M* = 0.45, *SD* = 0.42) than Indonesian books (*M* = 0.36, *SD* = 0.43), which in turn depicted more Internal challenge events than Japanese books (*M* = 0.18, *SD* = 0.27). Further analysis showed that the contrast between means for American vs. Japanese books approached significance (*p* = 0.079).

In addition, the proportion of solutions to challenge events that were coded as Individual in nature was obtained for each book. A One-Way ANOVA on this measure showed a significant effect of country, *F*_(2, 59)_ = 3.62, *p* = 0.033. *Post-hoc* analyses showed that Japanese storybooks had a greater proportion of Individual solutions to problems compared to American books (*M*_Japanese_ = 0.77, *SD* = 0.36; *M*_American_= 0.44, *SD* = 0.43, *p* = 0.025). The mean proportion of Individual solutions for Indonesian books (*M* = 0.61, *SD* = 0.37) was not significantly different from the two other groups.

### Outcomes of challenges

We hypothesized that collectivist vs. individualist themes might be revealed by the types of outcomes evidenced in the resolution of the overall dilemma or challenge presented in the narratives being examined. First, the overwhelming majority of books depicted successful resolution of the overall challenge depicted in the book. Only 2 or fewer out of the 20 books in each country presented a failure to achieve success. For the successes, outcomes coded as having an Individual, Other-oriented, or Shared (between self and other) benefit were analyzed to see if patterns varied by country. A chi-square test showed no significant differences in how these outcome scores were distributed across the three countries, χ^2^_(4)_ = 6.00, *p* = 0.199. Most outcomes were directed toward the main characters themselves or shared between main characters and others. In no instance were others the sole beneficiaries of the problem resolution.

## Discussion

The current study examined storybooks targeted for preschoolers from Indonesia, Japan, and the United States for the presence of achievement-oriented themes. The books were analyzed in terms of the number of challenge events, the source of each challenge, and the type of solution for the challenge. The results revealed several interesting findings, as discussed below.

First, while Japanese storybooks were similar to American storybooks in the number of challenge events, these groups differed in terms of the source of the challenge and the solution for the challenge. The challenges depicted in American storybooks tended to be due to internal qualities of the main character, but the solutions for the challenge were mostly external in nature (i.e., the main character typically received help from others to overcome the challenge). The somewhat greater emphasis on internal sources of challenge in American storybooks is consistent with previous observations that American children tend to exhibit ability beliefs (vs. effort beliefs) (Holloway, 1988; Stevenson and Stigler, 1992). In contrast, challenge events in Japanese storybooks were mostly caused by external factors, but the solutions were mostly individual in nature. This emphasis on individual effort complements Heine et al.'s (2001) observations that Japanese students were more likely to persevere during challenging tasks compared to American students. Moreover, the Japanese storybooks' emphasis on individual solutions is particularly interesting when we consider the higher tendency for the challenge to be externally caused. It seems that in addition to effort and perseverance—values that have been observed in Japanese parenting (Holloway et al., 1986)—Japanese storybooks also convey a message of personal responsibility. That is, regardless of the source of the challenge, Japanese children are receiving the message to take “ownership” of the problem by exerting individual effort to find a solution. This pattern of findings is consistent with the idea that in Japanese society, notions of self are malleable and individuals are expected to focus on self-improvement (Heine et al., 2001).

Second, compared to the American and Japanese books, the Indonesian storybooks depicted the fewest number of challenge events. This pattern may indicate a smaller cultural emphasis on meeting and overcoming challenges. It also seems to complement previous observations that Indonesian students focus on externally defined performance goals instead of mastery goals (Liem and Nie, 2008), since mastery goals often require individual effort and perseverance. Of the challenge events analyzed, Indonesian storybooks fell in the middle between American and Japanese storybooks in terms of the source of the challenge (Internal vs. External) and the solution for the challenge (Individual vs. Social). This pattern of findings suggests that it might be premature or even inappropriate to make broad, sweeping assumptions about how achievement values align with collectivist vs. individualist tendencies. There may be more nuanced ways in which beliefs about effort interface with cultural values, whether collectivist or individualist, perhaps depending on other social forces such as religion, political history, or economic circumstances. Indeed, cultural orientation and achievement beliefs may even be orthogonal constructs. Certainly, these are rich areas for future research.

In conclusion, the present findings suggest that as cultural products, storybooks do seem to convey to young children some important, culturally valued messages about effort and achievement. Future work should examine whether and how values of effort and perseverance are conveyed in storybooks targeted for older age groups. As children get closer to formal schooling, do storybooks help prepare them by making these values more explicit compared to books for younger ages? It would be important to look for messages about schooling and in particular, whether schooling and its associated activities are described in terms of obligations (“work”) or more enjoyable “opportunities to learn.” More detailed analyses on the language by which these messages are framed could be informative.

### Conflict of interest statement

The authors declare that the research was conducted in the absence of any commercial or financial relationships that could be construed as a potential conflict of interest.

## References

[B1] Bandura A. (1997). Self-Efficacy: The Exercise of Control. New York, NY: Freeman

[B2] Blackwell L. S. Trzesniewski K. H. Dweck C. S. (2007). Implicit theories of intelligence predict achievement across an adolescent transition: a longitudinal study and an intervention. Child Dev. 78, 246–263 10.1111/j.1467-8624.2007.00995.x 17328703

[B3] Duckworth A. L. Peterson C. Matthews M. D. Kelly D. (2007). Grit: Perseverance and passion for long-term goals. J. Pers. Soc. Psychol. 92, 1087–1011 10.1037/0022-3514.92.6.1087 17547490

[B4] Duckworth A. L. Seligman M. E. P. (2005). Self-discipline outdoes IQ in predicting academic performance of adolescents. Psychol. Sci. 16, 939–944 10.1111/j.1467-9280.2005.01641.x 16313657

[B5] Dweck C. S. (1999). Self-Theories: Their Role in Motivation. Ann Arbor, MI: Psychology Press

[B6] Dyer J. R. Shatz M. Wellman H. M. (2000). Young children's storybooks as a source of mental state information. Cogn. Dev. 15, 17–37 10.1016/S0885-2014(00)00017-4

[B7] Dyer-Seymour J. R. Shatz M. Wellman H. M. Saito M. T. (2004). Mental state expression in US and Japanese children's books. Int. J. Behav. Dev. 28, 546–552 10.1080/01650250444000261

[B8] Elliot A. J. McGregor H. A. Gable S. (1999). Achievement goals, study strategies, and exam performance: a meditational analysis. J. Educ. Psychol. 91, 549–563 10.1037/0022-0663.91.3.549

[B9] Heine S. J. (2001). Self as cultural product: an examination of East Asian and North American selves. J. Pers. 69, 881–906 10.1111/1467-6494.696168 11767822

[B10] Heine S. J. Kitayama S. Lehman D. R. Takata T. Ide E. Leung C. (2001). Divergent consequences of success and failure in Japan and North America: An investigation of self-improving motivations and malleable selves. J. Pers. Soc. Psychol. 81, 599–615 10.1037/0022-3514.81.4.599 11642348

[B11] Hofstede G. (1991). Cultures and Organizations: Software of the Mind. London: McGraw-Hill

[B12] Holloway S. D. (1988). Concepts of ability and effort in Japan and the United States. Rev. Educ. Res. 58, 327–345 10.3102/00346543058003327

[B13] Holloway S. D. Kashiwagi K. Hess R. D. Azuma H. (1986). Causal attributions by Japanese and American mothers about performance in mathematics. Int. J. Psychol. 21, 269–286 10.1080/00207598608247590

[B14] Lamoreaux M. Morling B. (2012). Outside the head and outside individualism-collectivism: further analysis of cultural products. J. Cross Cult. Psychol. 43, 299–327 10.1177/002202211038523418544712

[B15] Liem A. D. Nie Y. (2008). Values, achievement goals, and individual-oriented and social-oriented achievement motivations among Chinese and Indonesian secondary school students. Int. J. Psychol. 43, 898–903 10.1080/00207590701838097 22022795

[B17] Sensus Penduduk (2010). Jakarta. Indonesia: Badan Pusat Statistik

[B18] Skinner E. A. Wellborn J. G. Connell J. P. (1990). What it takes to do well in school and whether I've got it: a process model of perceived control and children's engagement and achievement in school. J. Educ. Psychol. 82, 22–32 10.1037/0022-0663.82.1.22

[B19] Stevenson H. W. Stigler J. W. (1992). The Learning Gap. New York, NY: Summit Books

[B20] Tsai J. L. Louie J. Y. Chen E. E. Uchida Y. (2007). Learning what feelings to desire: socialization of ideal affect through children's storybooks. Pers. Soc. Psychol. Bull. 33, 17–30 10.1177/0146167206292749 17178927

[B21] Ziegert D. I. Kistner J. A. Castro R. Robertson B. (2001). Longitudinal study of young children's responses to challenging achievement situations. Child Dev. 72, 609–624 10.1111/1467-8624.00300 11333088

[B22] Zimmerman B. J. (2000). Self-efficacy: an essential motive to learn. Contemp. Educ. Psychol. 25, 82–91 10.1006/ceps.1999.1016 10620383

