# Supplementary material for: “I think I can”: achievement-oriented themes in storybooks from Indonesia, Japan, and the United States
Source: Front Psychol. 2014 Mar 4;5:167. doi: 10.3389/fpsyg.2014.00167 (PMC3941212; doi:10.3389/fpsyg.2014.00167)
Supplement: Supplementary file 1 [file DataSheet1.DOCX]

**Supplemental Material:** Complete list of storybooks included in Suprawati, Anggoro, & Bukatko

| Indonesia | Japan | United States |
| --- | --- | --- |
| Akibat Buang Sampah Sembarangan | Guri to Gura no Okyaku Sama | How My Garden Grew |
| Berkat Dua Ekor Anak Ayam | Kasajizou | Henny Penny |
| Beruang dan Kupu-Kupu | Sentaku Kaachan | Clifford the Big Red Dog |
| Dodo dan Caca | Kobujii Sama | Gone Fishing |
| Domba dan Serigala | Watashi no Wanpiisu | Play with Me |
| Kiki Tikus | Haketa yo Haketa yo | Swimmy |
| Kuda Pak Ogi | Tarou no Odekake | The Blue Ribbon Puppies |
| Kumbang yang Sombong | Zou Kun no Sanpo | The Golden Egg Book |
| Musim Hujan | Tokochan wa Doko | Faye and Dolores |
| Petualangan Anak Laut | Shoubou Jidousha Jiputa | Alfie Gives a Hand |
| Pergi ke Kebun Binatang | Guri to Gura | In the Night Kitchen |
| Piko si Pemalas | Noroma na Rooraa | Sometimes I'm Afraid |
| Rajin Menabung | Darumachan to Tenguchan | Will I Have a Friend? |
| Saling Memaafkan | Issun Boushi | The Mixed-Up Chameleon |
| Semut dan Merpati | Sakasama Raion | The Runaway Bunny |
| Si Belang Temanku | Nezumikun no Chokki | Pierre |
| Suara Apa Itu? | Boku no Kureyon | The Little Engine That Could |
| Todi dan Semut | Kaba Kun | Danny and the Dinosaur |
| Ulat menjadi Kupu-Kupu | Sora Iro no Tane | Barkley |
| Wooly Pergi Berlayar | Gurunpa no Youchien | Addie Meets Max |
